# Development and feasibility testing of an AI-powered chatbot for early detection of caregiver burden: protocol for a mixed methods feasibility study

**DOI:** 10.3389/fpsyt.2025.1553494

**Published:** 2025-02-28

**Authors:** Ravi Shankar, Anjali Bundele, Amanda Yap, Amartya Mukhopadhyay

**Affiliations:** ^1^ Research and Innovation, Medical Affairs, Alexandra Hospital, Singapore, Singapore; ^2^ Division of Respiratory and Critical Care Medicine, Department of Medicine, National University Hospital, Singapore, Singapore

**Keywords:** caregiver burden, artificial intelligence, chatbot, natural language processing, chronic conditions, feasibility study

## Abstract

**Introduction:**

Caregivers of patients with end-stage kidney disease (ESKD) face significant challenges that contribute to caregiver burden, negatively impacting their physical, psychological, social, and financial well-being. With the growing prevalence of chronic diseases and an aging population, there is an urgent need for accessible and scalable solutions to detect and address caregiver burden. Artificial Intelligence (AI) chatbots using natural language processing (NLP) have shown promise in providing mental health support and monitoring through natural conversations. This study will contribute to research and clinical practice by: (1) validating a novel approach for early detection of caregiver burden through NLP, (2) analyzing the feasibility of AI-powered chatbots for continuous caregiver monitoring, and (3) informing the development of scalable, accessible tools to identify at-risk caregivers.

**Methods and analysis:**

This protocol for the mixed methods aims to evaluate the feasibility, acceptability, and preliminary effectiveness of BOTANIC (Burden Observation and Timely Aid for Navigating Informal Caregiving), an AI-powered chatbot for early detection of caregiver burden. A single-center validation study will be conducted at Alexandra Hospital, Singapore. Twenty primary caregivers of ESKD patients will be recruited to use BOTANIC for 12 weeks. BOTANIC, developed using Python and open-source libraries, will integrate with Telegram and utilize advanced NLP techniques to analyze caregiver conversations and detect signs of burden. The NLP algorithm will analyze conversations to generate burden scores at baseline and at 12 weeks. Participants will also complete baseline and 12-week assessments using validated questionnaires including the Zarit Burden Interview (ZBI), Patient Health Questionnaire-9 (PHQ-9), and Generalized Anxiety Disorder-7 (GAD-7). Primary outcomes include concordance between caregiver burden levels detected by the NLP algorithm and validated assessment scores at both timepoints. Secondary outcomes include user engagement metrics and system satisfaction. Semi-structured interviews will explore participants’ experiences with the chatbot. Quantitative data will be analyzed using descriptive statistics and appropriate statistical tests such as paired t-tests or Wilcoxon signed-rank tests, while qualitative data will undergo thematic analysis.

**Ethics and dissemination:**

The study has been approved by the NHG Domain Specific Review Board. Findings will be published in peer-reviewed journals, presented at conferences, and used to inform the development of larger-scale trials of AI-powered caregiver support interventions.

## Background

Caregiver burden is defined as the multidimensional response to the physical, psychological, social, and financial stressors associated with caregiving (1, 2). With the increasing prevalence of chronic diseases and an aging population, the demand for informal caregiving by caregivers such as family members is on the rise (3, 4). Caregivers play a crucial role in supporting individuals with various chronic conditions, such as dementia, cancer, stroke, and end-stage kidney disease, across different care settings, including home, community, and healthcare facilities (5).

Despite the vital contributions of informal caregivers to the healthcare system and society, caregiving often comes at a significant personal cost. Caregivers are at increased risk of physical health problems, such as cardiovascular disease, immune dysfunction, and premature mortality (6). They also experience high rates of psychological distress, including depression, anxiety, and burnout (7). Moreover, caregiving can lead to social isolation, financial strain, and reduced quality of life (8). These negative consequences, collectively known as caregiver burden, can have far-reaching impacts on caregivers, care recipients, and the healthcare system as a whole.

Current approaches to assessing and addressing caregiver burden have several limitations. Traditional assessment methods, such as the Zarit Burden Interview (ZBI) (9) and the Caregiver Strain Index (CSI) (10), rely on infrequent self-report measures that may not capture the dynamic nature and contextual factors influencing caregiver burden. These assessments are often administered in clinical settings, which can be time-consuming, resource-intensive, and may not reflect caregivers’ experiences in real-world settings. Moreover, the lack of regular monitoring and timely support can lead to the escalation of caregiver burden over time, resulting in adverse outcomes such as caregiver burnout, poor mental health, and decreased quality of care (11).

To address these challenges, there is a growing interest in leveraging digital health technologies, particularly artificial intelligence (AI) chatbots, for caregiver support. AI chatbots are computer programs that simulate human conversation using natural language processing (NLP) techniques (12). NLP enables computers to understand, interpret, and generate human language, allowing for more natural and engaging interactions between users and chatbots (13). By analyzing the linguistic and semantic features of caregivers’ responses during chatbot conversations, NLP algorithms can potentially detect signs of caregiver burden, provide personalized support, and facilitate early intervention (14).

Recent studies have demonstrated the significant potential of AI chatbots for mental health support, screening, and behavior change interventions (15–22). Multiple chatbots using different therapeutic approaches have shown promising results. For example, Woebot, a conversational agent based on cognitive-behavioral therapy principles, reduced symptoms of depression and anxiety among college students and demonstrated high engagement rates (15). Wysa, an AI-powered mental health chatbot using a combination of CBT, dialectical behavior therapy, and motivational interviewing techniques, showed improvements in depression, anxiety, and sleep outcomes (16). Similarly, Ai-driven Tess provided on-demand support and coping strategies for individuals with mental health concerns through empathetic responses and demonstrated good feasibility and acceptability (17). Anna, an AI chatbot by Happify Health, successfully increased user engagement in digital mental health interventions through personalized interactions (18). DigiQuit showed success in smoking cessation through personalized behavioral support (19), while CASC demonstrated efficacy in medication adherence monitoring (20).

Studies have shown chatbots can successfully increase adherence to healthy behaviors through theory-based approaches including goal setting, monitoring, real-time reinforcement/feedback, and on-demand support (21). Results demonstrate particular efficacy in promoting healthy lifestyles (40% of studies), smoking cessation (27%), treatment/medication adherence (13%), and reduction in substance misuse (7%) (22).

While AI chatbots have been widely studied for mental health support and monitoring, their specific application to caregiver burden detection and support remains largely unexplored. Previous technological approaches to addressing caregiver burden have primarily focused on digital support groups, educational resources, and care coordination tools. To our knowledge, this is the first study to evaluate an AI-powered chatbot specifically designed for early detection and monitoring of caregiver burden through natural language conversations.

In the context of caregiving, AI chatbots offer several potential advantages over traditional support methods. First, chatbots can provide 24/7 availability and accessibility, allowing caregivers to seek support at their convenience and in the comfort of their own homes (21, 23). Second, the anonymity and non-judgmental nature of chatbots may create a safe space for users to disclose sensitive information and experiences more openly, overcoming the stigma and barriers associated with seeking help (24, 25). Third, chatbots can deliver personalized and tailored support based on caregivers’ individual needs, preferences, and context, using machine learning algorithms to adapt and optimize the conversation flow (26). Fourth, their integration into commonly used platforms like smartphones and messaging apps enables them to reach large and diverse populations (27).

Building on this foundation, we propose to develop and evaluate BOTANIC (Burden Observation and Timely Aid for Navigating Informal Caregiving), an AI chatbot system designed to detect caregiver burden among informal caregivers of patients with various chronic conditions. BOTANIC will leverage advanced NLP techniques and machine learning algorithms to analyze caregivers’ responses during chatbot conversations and identify linguistic and behavioral markers of burden. By enabling early detection and continuous monitoring through natural language conversations, BOTANIC aims to help healthcare providers better understand and track caregiver burden patterns over time, potentially informing more timely and appropriate support interventions.

The specific aims of the feasibility study will be:

1. To develop BOTANIC, a Python-based AI chatbot system that uses NLP and machine learning to detect signs of caregiver burden through conversations with caregivers.
2. To assess the preliminary effectiveness of BOTANIC in detecting caregiver burden, as measured by the NLP algorithm and validated caregiver-reported outcome measures, and to explore its impact on secondary outcomes, including user engagement, system satisfaction, and caregiver well-being (e.g., depression, anxiety, quality of life).
3. To evaluate the feasibility and acceptability of BOTANIC among informal caregivers of patients with various chronic conditions at a tertiary public hospital through a validation study design.
4. To gather qualitative feedback from caregivers on their experiences with BOTANIC, perceived benefits and challenges, and suggestions for future enhancements.

We hypothesize that BOTANIC will show promising results in detecting caregiver burden, with significant correlation between NLP-detected burden levels and caregiver-reported outcomes at baseline and follow-up. We also expect BOTANIC to demonstrate feasibility and acceptability among informal caregivers, as indicated by high user engagement, system satisfaction, and positive qualitative feedback.

This paper presents the protocol for developing and evaluating BOTANIC, detailing the planned methodology for both the technical development and feasibility testing phases. No results are presented as this is a protocol paper describing the proposed study design and methods.

## Methods

### Study design

This study will employ a concurrent validation design to evaluate BOTANIC’s burden detection capabilities among informal caregivers of patients with various chronic conditions, using a mixed-methods approach at a tertiary public hospital in Singapore. The study will consist of two main phases: (1) Development of BOTANIC Chatbot, and (2) feasibility testing, using quantitative surveys and qualitative interviews.

### Phase 1: development of BOTANIC

#### Foundational model selection and fine-tuning

For the development of BOTANIC, we will leverage a state-of-the-art foundational large language model (OpenAI’s GPT-4), to analyze caregiver responses and detect signs of caregiver burden. The selection of this foundational model is based on factors such as performance, flexibility, and cost-effectiveness for our caregiver chatbot application. Subsequently, we will fine-tune the model using a curated dataset of caregiver conversations and annotations, focusing on specific questions and topics related to caregiver burden. This dataset will be sourced from various online forums, support groups, and existing caregiver chatbot systems, and will be annotated to identify linguistic and semantic patterns associated with caregiver burden. The fine-tuning process will involve adapting the pre-trained language model to the specific domain and style of caregiver conversations, as well as optimizing its performance for the task of caregiver burden detection based on the carefully designed questions.

#### Telegram bot development

BOTANIC will be developed as a Telegram bot using the Python programming language and the python-telegram-bot library. This library provides a simple and intuitive interface for building chatbots on the Telegram platform, allowing BOTANIC to engage with caregivers through natural conversational interactions. Unlike traditional structured questionnaires, caregivers can express their experiences, feelings, and concerns in their own words through free-text messaging, while the system’s underlying architecture processes these natural responses. The bot’s questions are carefully designed based on the Zarit Burden Interview, a validated instrument for detecting caregiver burden. The questions are organized into key domains that research has shown to be critical indicators of caregiver burden:

1. Physical and emotional demands of caregiving - These questions assess the direct impact of caregiving activities and responsibilities on the caregiver’s wellbeing, which research shows is a primary indicator of burden levels.
2. Impact on personal relationships and social life - Questions examining how caregiving affects relationships with family and friends, as social isolation is a significant contributor to caregiver burden.
3. Financial strain associated with caregiving - Research demonstrates that economic pressures from caregiving responsibilities are a major source of burden, particularly for those caring for patients with chronic conditions.
4. Expectations and sense of duty - These questions evaluate feelings of obligation and perceived expectations from others, which the Zarit Burden Interview identifies as key burden indicators.
5. Personal life impact and self-care - Assessment of how caregiving affects the caregiver’s ability to maintain their own life and wellbeing, which research shows directly correlates with burden levels.

This assessment framework implements the Zarit Burden Interview through natural conversation, with additional validated measures (PHQ-9, GAD-7) included as supplementary indicators to help validate burden detection. The questions are designed to gather burden indicators while maintaining a supportive dialogue that encourages honest responses. Each category’s questions are introduced contextually to maintain conversation flow while ensuring systematic assessment of burden dimensions.

The NLP algorithm will employ a multi-stage approach to detect caregiver burden:

1. Text preprocessing including tokenization, lemmatization, and removal of stop words.
2. Feature extraction focusing on linguistic markers associated with burden (emotional words, stress indicators, fatigue-related terms).
3. Semantic analysis using GPT-4’s embeddings to understand context and emotional content.
4. Pattern recognition across multiple conversations to identify trends in burden indicators.
5. Integration of structured assessment responses (ZBI questions) with unstructured conversation analysis.
6. Generation of a continuous burden score using a weighted combination of these features.

GPT-4 will serve two distinct functions: handling natural conversation flow and supporting burden detection through semantic analysis. The conversational aspects will be managed through GPT-4’s dialogue capabilities, while a separate custom NLP pipeline will analyze the conversations for burden indicators, using GPT-4’s embeddings as one component of the analysis.

Unlike traditional digital questionnaires, BOTANIC is designed to provide a more engaging and naturalistic interaction experience through conversational AI. The chatbot will offer several advantages over standard digital forms: 1) ability to ask follow-up questions based on user responses, 2) detection of subtle linguistic markers of burden that may not be captured in structured questionnaires, 3) provision of empathetic responses and supportive interactions, and 4) ongoing monitoring through regular conversations rather than point-in-time assessments. Additionally, BOTANIC will provide educational resources about caregiving, stress management techniques, and links to support services based on detected burden levels.

The bot will use the fine-tuned foundational model to analyze the caregiver’s responses and detect potential indicators of caregiver burden. The conversation flow will be structured to gather relevant information and provide a safe space for caregivers to express their experiences and feelings. The bot will be designed to be empathetic and understanding while focusing on the primary goal of detecting caregiver burden. We will implement error handling and fallback mechanisms to ensure that the bot can gracefully handle unexpected user inputs or technical issues, and provide appropriate guidance and support to the caregiver throughout the conversation.

#### Testing and evaluation

Throughout the development process, we will conduct rigorous testing and evaluation to ensure the quality, reliability, and effectiveness of BOTANIC. This will involve unit testing and integration testing of the bot’s functionality, as well as performance testing to ensure it can handle a large number of concurrent users and conversations. We will also conduct user acceptance testing (UAT) with a diverse group of caregivers to gather feedback on the bot’s usability, clarity of questions, and overall user experience. This feedback will be used to identify areas for improvement and to iteratively refine the bot’s performance.

### Phase 2: feasibility testing

#### Participants

A purposive sample of 20 primary informal caregivers of patients with various chronic conditions will be recruited from the outpatient clinics, inpatient wards, and community services affiliated with Alexandra Hospital. The inclusion criteria are: (1) aged 21 years or above, (2) providing primary care for a patient with a chronic condition (e.g., dementia, cancer, stroke, heart disease, end-stage kidney disease) for at least 3 months, (3) able to communicate in English, (4) owning a smartphone with a compatible messaging app installed, and (5) willing to use BOTANIC for 12 weeks. Caregivers with severe cognitive impairment, active psychosis, or substance abuse will be excluded. A sample size of 20 is considered sufficient for feasibility testing, allowing for a diverse representation of caregivers and chronic conditions (28–30).

#### Procedures

Eligible caregivers will be approached by a research coordinator during their care recipients’ clinic visits, hospital stays, or community events. The study purpose, procedures, risks, and benefits will be explained, and written informed consent will be obtained. Participants will complete a baseline assessment, including demographic information, the Zarit Burden Interview (ZBI), the Patient Health Questionnaire-9 (PHQ-9), and the Generalized Anxiety Disorder-7 (GAD-7). They will then receive training on how to use BOTANIC and be encouraged to engage with the chatbot regularly throughout the 12-week study period. The NLP algorithm will analyze initial conversations to establish baseline burden scores, and continue analyzing conversations throughout the study period, with final burden scores calculated at week 12. The validated assessment questionnaires (ZBI, PHQ-9, GAD-7) will also be repeated at 12 weeks. This will allow comparison between the NLP-detected burden levels and standard assessment scores at both timepoints.

#### Outcomes

The primary outcome is the concordance between burden levels detected by BOTANIC’s NLP algorithm and standard assessment measures (ZBI, PHQ-9, GAD-7). The algorithm will assign a continuous burden score based on the linguistic and semantic features of caregivers’ responses, with higher scores indicating greater burden severity. Secondary outcomes include user engagement metrics (conversation frequency, duration, response rate), and system usability and satisfaction (System Usability Scale, User Experience Questionnaire).

#### Data analysis

Descriptive statistics will be used to summarize participants’ demographic and clinical characteristics, engagement metrics, and usability and satisfaction ratings. Means, standard deviations, and proportions will be reported as appropriate. The feasibility and acceptability of BOTANIC will be evaluated based on predefined criteria, such as the proportion of eligible caregivers enrolled, the proportion of participants completing the study, average engagement levels, and mean usability and satisfaction scores.

The preliminary effectiveness of BOTANIC in detecting caregiver burden will be assessed using paired t-tests or Wilcoxon signed-rank tests, comparing the NLP-generated burden scores and ZBI, PHQ-9, GAD-7 scores at baseline and 12 weeks. Effect sizes (Cohen’s d) will be calculated to determine the magnitude of change.

To evaluate the bot’s effectiveness in detecting caregiver burden, we will employ both quantitative and qualitative methods. Quantitatively, we will analyze the caregiver responses to the bot’s questions and compare the detected burden levels with the ZBI scores. This will help us assess the accuracy and validity of the bot’s burden detection capabilities. Qualitatively, we will conduct interviews with caregivers who have interacted with the bot to gather their feedback on the relevance and clarity of the questions, the bot’s ability to understand their experiences, and the overall user experience of interacting with the bot.

The caregivers who completed the feasibility testing phase will be invited to participate in semi-structured interviews. Participants will be selected to achieve maximum variation in terms of demographic characteristics, chronic conditions, caregiving experiences, engagement levels, and changes in caregiver burden scores. Recruitment will continue until data saturation is reached, i.e., no new themes or insights emerge from additional interviews.

#### Qualitative data collection

Individual interviews will be conducted by a trained qualitative researcher using a semi-structured interview guide. The guide will explore caregivers’ motivations for using BOTANIC, perceived benefits and challenges, impact on caregiving experiences and well-being, and suggestions for improving the chatbot’s content, functionality, and user experience. Interviews will be conducted in English, either in-person or via videoconferencing, based on participants’ preferences and public health guidelines. Interviews will be audio-recorded, transcribed verbatim, and anonymized. Field notes will be taken to capture non-verbal cues, contextual information, and researcher reflections.

The qualitative component of this study will follow the Consolidated Criteria for Reporting Qualitative Studies (COREQ) guidelines to ensure comprehensive and transparent reporting of qualitative research findings.

#### Thematic analysis

The interview transcripts will be analyzed using thematic analysis, following Braun and Clarke’s six-phase approach (31). The analysis will involve familiarization with the data, generating initial codes, searching for themes, reviewing and refining themes, defining and naming themes, and producing the final report. The analysis will be conducted iteratively, with constant comparison within and across cases, to ensure the coherence and distinctiveness of the themes. Coding will be performed independently by two researchers, with regular meetings to discuss and resolve discrepancies. The qualitative findings will be integrated with the quantitative results using a mixed-methods matrix (32), facilitating a comprehensive understanding of the feasibility, acceptability, and impact of BOTANIC from the caregivers’ perspectives.

**Figure 1:**
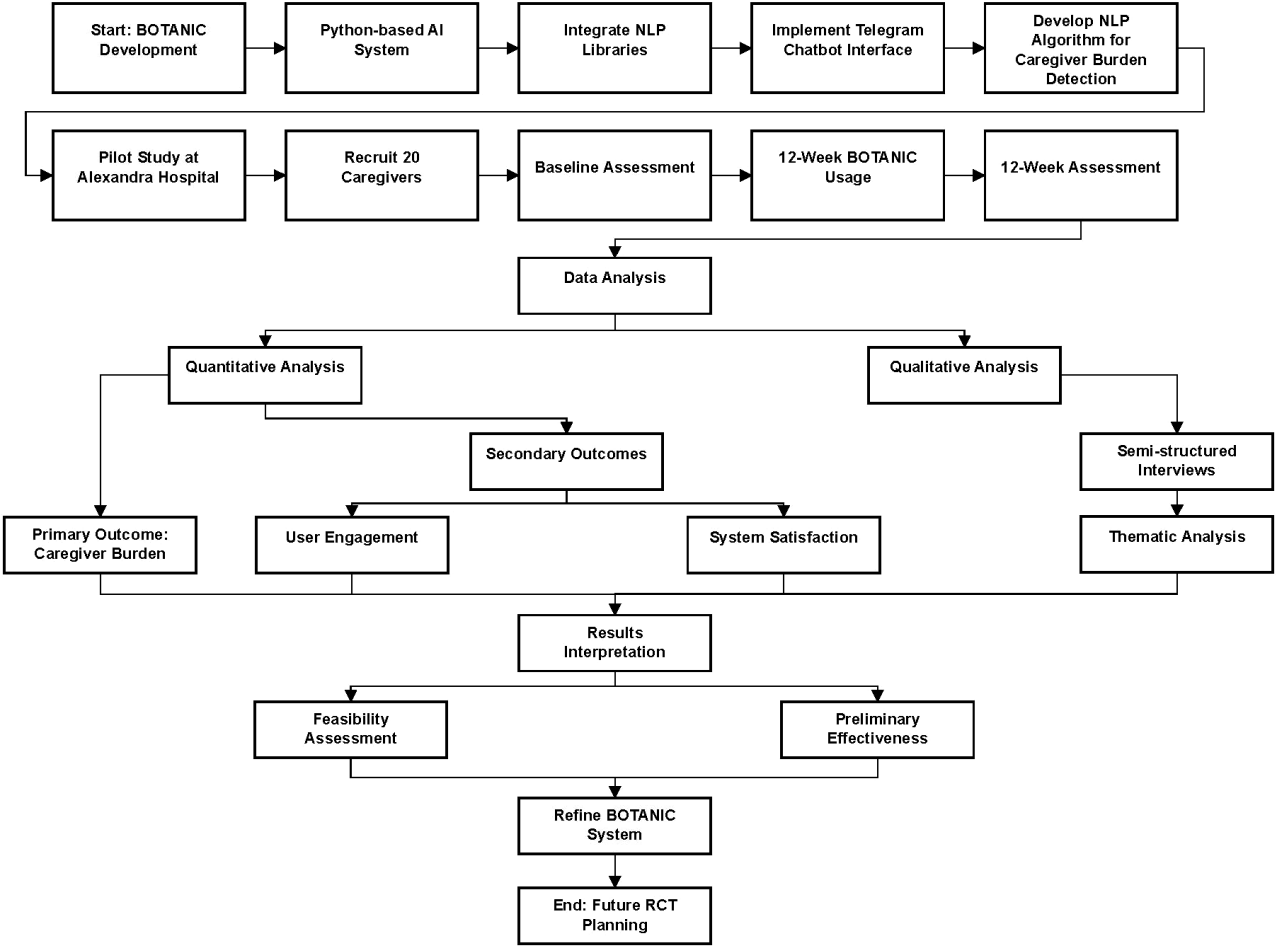
Study flow diagram showing the two main phases.

**Figure 1** presents the study flow diagram illustrating both the technical development of BOTANIC and its clinical feasibility testing pathway.

### Patient and public involvement

Informal caregivers contributed to this study’s conceptualization and design. During development, two caregiver representatives assessed intervention feasibility and helped refine outcome measures. These representatives will participate in results interpretation. Study findings will be disseminated to participants through written summaries and a presentation session.

## Discussion

The proposed study aims to evaluate the feasibility, acceptability, and preliminary effectiveness of BOTANIC, an AI-powered chatbot for early detection of caregiver burden. If successful, BOTANIC could provide a scalable and accessible solution to address the growing issue of caregiver burden across various chronic conditions and care settings.

The study’s strengths lie in its innovative approach, combining advanced NLP techniques and machine learning algorithms to analyze caregiver conversations and detect signs of burden. The NLP algorithm employs a multi-stage approach that includes text preprocessing, feature extraction of linguistic burden markers, semantic analysis using GPT-4’s embeddings, pattern recognition across conversations, integration of structured assessments, and generation of continuous burden scores. GPT-4 serves dual functions - managing natural conversations while supporting burden detection through semantic analysis, with a separate NLP pipeline analyzing conversations for burden indicators. This approach differs from previous chatbot interventions, which have primarily focused on providing general mental health support or adherence to specific health behaviors (15–22). By tailoring the chatbot’s content and functionality to the specific needs and experiences of caregivers, BOTANIC has the potential to offer more targeted and personalized support. The mixed-methods design, incorporating both quantitative and qualitative data, will provide a comprehensive understanding of the chatbot’s performance, user experience, and impact on caregiver outcomes.

The study’s focus on a diverse sample of caregivers in the multi-racial Singaporean context is another strength, as it will provide insights into the chatbot’s acceptability and effectiveness across different cultural and linguistic backgrounds. This is particularly important given the growing recognition of the need for culturally sensitive and inclusive caregiver interventions (33, 34). The study’s findings will contribute to the limited literature on digital health interventions for caregiver support in Asian populations and inform the adaptation of BOTANIC for other cultural contexts.

However, several limitations should be acknowledged. The small sample size (n=20) and single-center design may limit the generalizability of the findings to wider caregiver populations and healthcare settings. The 12-week study duration, while sufficient for feasibility testing, may not capture the long-term engagement, effectiveness, and sustainability of the chatbot. Future studies should include larger, multi-center samples and longer follow-up periods to establish the robustness and scalability of BOTANIC.

The effectiveness of BOTANIC will largely depend on the quality and diversity of the training data used to develop the NLP models. Ensuring the representativeness, validity, and cultural sensitivity of the data is crucial to avoid bias and improve the chatbot’s performance across different caregiver populations and contexts. Collaboration with caregivers, healthcare professionals, and community organizations will be essential to create large, diverse, and high-quality datasets that capture the complexity and nuances of caregiver experiences.

Another critical consideration is the accuracy and reliability of the algorithm. Rigorous testing, validation, and continuous monitoring of the NLP models and dialogue management system will be necessary to ensure the chatbot’s performance, consistency, and appropriateness. Establishing clear protocols for handling high-risk situations and integrating human oversight and escalation mechanisms will be essential to ensure the chatbot’s safety and effectiveness.

The successful implementation of BOTANIC in real-world settings will require careful consideration of the technical, organizational, and human factors that influence its adoption, usability, and sustainability. Integration with existing healthcare systems, such as electronic health records and caregiver support services, will be necessary to ensure seamless data sharing, care coordination, and continuity of support. Engaging key stakeholders, including caregivers, healthcare professionals, administrators, and policymakers, in the co-design, evaluation, and dissemination of the chatbot will be crucial to ensure its relevance, acceptability, and alignment with their needs and priorities.

Ethical and social implications of using AI chatbots for caregiver support should also be thoroughly examined and addressed. Ensuring data privacy, security, and confidentiality is paramount to protect caregivers’ sensitive information and maintain their trust in the system. Obtaining informed consent, respecting caregivers’ autonomy and preferences, and providing clear information about the chatbot’s capabilities and limitations are essential to ensure ethical and transparent use of the technology.

Future research should focus on larger, multi-center trials with longer follow-up periods to establish the effectiveness, cost-effectiveness, and sustainability of BOTANIC. Economic evaluations, such as cost-utility and cost-benefit analyses, will be necessary to demonstrate the value proposition and return on investment of the chatbot compared to usual care or alternative interventions.

## Conclusion

This protocol describes the development and planned evaluation of an AI-powered chatbot system (BOTANIC) designed to detect and monitor caregiver burden through natural conversations. BOTANIC represents an innovative approach to address the urgent need for accessible and scalable solutions to detect and support caregivers at risk of burden. By harnessing AI and NLP technologies, BOTANIC aims to provide personalized and timely support to caregivers through natural conversations, potentially improving their well-being and the quality of care they provide.

This feasibility study is a crucial first step in evaluating the potential of BOTANIC and informing its future development and implementation. The findings will contribute to the evidence base on digital health interventions for caregiver support and guide the design of larger, definitive trials.

However, the success of BOTANIC will ultimately depend on its ability to accurately detect burden, provide safe and appropriate support, and seamlessly integrate with existing healthcare systems and caregiver support services. Engaging caregivers, healthcare professionals, and other stakeholders in the co-design and evaluation process will be essential to ensure the chatbot’s relevance, acceptability, and adoption in real-world settings.

As the global burden of chronic diseases and the demand for informal caregiving continue to rise, innovative solutions like BOTANIC offer hope for a more sustainable and compassionate model of caregiver support. With further research and refinement, AI-powered chatbots have the potential to transform the landscape of caregiver interventions and contribute to better outcomes for caregivers, care recipients, and healthcare systems worldwide.
