# Supplementary material for: Development and feasibility testing of an AI-powered chatbot for early detection of caregiver burden: protocol for a mixed methods feasibility study
Source: Front Psychiatry. 2025 Feb 28;16:1553494. doi: 10.3389/fpsyt.2025.1553494 (PMC11907196; doi:10.3389/fpsyt.2025.1553494)
Supplement: Supplementary file 1 [file DataSheet1.docx]

**BOTANIC Study: Baseline Data Collection Form**

**Administrative Information**

Date: _______________ Participant ID: _______________ Research Staff ID: _______________

**A. Caregiver Demographics**

1. Age: _____ years
2. Gender: □ Male □ Female □ Other □ Prefer not to say
3. Ethnicity: □ Chinese □ Malay □ Indian □ Others (specify): _______________
4. Marital Status: □ Single □ Married □ Divorced/Separated □ Widowed
5. Education Level: □ Primary or below □ Secondary □ Post-secondary/Diploma □ University degree □ Postgraduate
6. Employment Status: □ Full-time □ Part-time □ Retired □ Unemployed □ Others (specify): _______________
7. Monthly Household Income (SGD): □ < $2,000 □ $2,000 - $4,999 □ $5,000 - $9,999 □ ≥ $10,000 □ Prefer not to say

**B. Caregiving Information**

1. Relationship to Care Recipient: □ Spouse □ Child □ Parent □ Sibling □ Other (specify): _______________
2. Duration of Caregiving: □ 3-6 months □ 7-12 months □ 1-2 years □ 2-5 years □ >5 years
3. Hours per week spent caregiving: □ <10 hours □ 10-20 hours □ 21-40 hours □ >40 hours
4. Do you have help from others? □ No □ Yes (specify): □ Other family members □ Domestic helper □ Professional caregivers □ Others: _______________

**C. Technology Usage**

1. Smartphone Ownership: □ Yes □ No
2. Telegram Usage Experience: □ Never used □ Occasional user □ Regular user □ Daily user
3. Comfort with Technology: □ Very uncomfortable □ Somewhat uncomfortable □ Neutral □ Somewhat comfortable □ Very comfortable

**D. Care Recipient Information**

1. Age: _____ years
2. Gender: □ Male □ Female
3. Duration of ESKD: □ <1 year □ 1-3 years □ 3-5 years □ >5 years
4. Current Treatment: □ Hemodialysis □ Peritoneal dialysis □ Conservative management
5. Comorbidities (check all that apply): □ Diabetes □ Hypertension □ Heart disease □ Others (specify): _______________

**E. Baseline Assessment Scores**

1. Zarit Burden Interview (ZBI) Score: _____
2. Patient Health Questionnaire-9 (PHQ-9) Score: _____
3. Generalized Anxiety Disorder-7 (GAD-7) Score: _____

**F. Additional Notes**

Completed by: _______________ Date completed: _______________
