# Supplementary material for: Development and feasibility testing of an AI-powered chatbot for early detection of caregiver burden: protocol for a mixed methods feasibility study
Source: Front Psychiatry. 2025 Feb 28;16:1553494. doi: 10.3389/fpsyt.2025.1553494 (PMC11907196; doi:10.3389/fpsyt.2025.1553494)
Supplement: Supplementary file 2 [file DataSheet2.docx]

**Participant Information Sheet and Consent Form**

**Study Title**

Development and feasibility testing of an AI-powered chatbot (BOTANIC) for early detection of caregiver burden: A mixed methods feasibility study

**Principal Investigator and Contact Details**

Dr. Ravi Shankar Research and Innovation, Medical Affairs Alexandra Hospital 378 Alexandra Road, Singapore 159964 Email: [Ravi_SHANKAR@nuhs.edu.sg](mailto:Ravi_SHANKAR@nuhs.edu.sg)

**Study Sponsor**

This study is funded by the National University of Singapore (NUS) Academic Health Programme (AHP) Fund (Reference: HPHSR-Enabling-Sep24/AHP/C01/RS).

**1. Introduction**

You are invited to participate in a research study. This form provides detailed information about the study. A member of the research team will describe this study to you and answer all of your questions. Please read the information below carefully and ask questions about anything you don't understand before deciding whether to take part in the study.

**2. Purpose of the Study**

The purpose of this study is to evaluate the feasibility, acceptability, and preliminary effectiveness of BOTANIC (Burden Observation and Timely Aid for Navigating Informal Caregiving), an AI-powered chatbot for early detection of caregiver burden among caregivers of patients with end-stage kidney disease (ESKD).

**3. Study Procedures**

If you agree to participate in this study, the following will occur:

a) **Screening**: You will be screened for eligibility based on the inclusion and exclusion criteria of the study.

b) **Initial Setup**:

- You will be provided with instructions on how to access BOTANIC through the Telegram messaging platform
- You will receive training on how to use the chatbot
- You will complete baseline assessments including:
  - Zarit Burden Interview (ZBI)
  - Patient Health Questionnaire-9 (PHQ-9)
  - Generalized Anxiety Disorder-7 (GAD-7)

c) **Intervention Period**:

- You will use BOTANIC for 12 weeks
- The chatbot will engage you in regular conversations to assess your caregiving experience
- You can interact with the chatbot at any time that is convenient for you
- The system will analyze your conversations to detect signs of caregiver burden

d) **Follow-up Assessments**:

- You will complete the same questionnaires (ZBI, PHQ-9, GAD-7) at week 12
- You may be invited to participate in a semi-structured interview about your experience with BOTANIC
- Interviews will be audio-recorded and transcribed for analysis

The total duration of your participation will be approximately 12 weeks.

**4. Risks and Discomforts**

The risks associated with this study are minimal. However, you may experience:

- Emotional discomfort when discussing your caregiving experiences
- Concerns about privacy when sharing information with the chatbot
- Time burden from engaging with the chatbot and completing assessments

If you experience any discomfort, you can pause your participation at any time and inform the research team. We will provide you with contact information for mental health support services if needed.

**5. Benefits**

You may or may not benefit directly from participating in this study. Potential benefits include:

- Early detection of caregiver burden through regular monitoring
- Access to a 24/7 platform for expressing your caregiving experiences
- Contributing to research that may help improve support for ESKD caregivers in the future

However, we cannot guarantee that you will experience these or any other benefits.

**6. Alternatives to Participation**

You do not have to participate in this study. Alternative options include:

- Continuing with your usual care and support services
- Seeking other forms of caregiver support outside of this study Your decision will not affect the care you or your loved one receives at Alexandra Hospital or any other healthcare facility.

**7. Confidentiality**

We will take the following measures to keep your personal information confidential:

- All data will be de-identified and stored securely on password-protected servers
- Conversations with BOTANIC will be encrypted and anonymized
- Only authorized research team members will have access to the data
- In any publication or presentation, you will not be identified by name
- Audio recordings of interviews will be transcribed and then destroyed
- Your data will be kept for 2 years after study conclusion and then securely destroyed

However, absolute confidentiality cannot be guaranteed. Your personal information may be disclosed if required by law.

**8. Costs and Compensation**

There is no cost to you for participating in this study. You will not receive any monetary compensation for participating.

**9. Voluntary Participation and Withdrawal**

Your participation is entirely voluntary. You can withdraw at any time without penalty. If you withdraw, you can request that your data be deleted, though anonymized data already analyzed may be retained.

**10. Questions and Contacts**

For study-related questions, contact: Dr. Ravi Shankar Email: [Ravi_SHANKAR@nuhs.edu.sg](mailto:Ravi_SHANKAR@nuhs.edu.sg)

For questions about your rights as a research participant, contact: National Healthcare Group Domain Specific Review Board (NHG DSRB) Research & Development Office Telephone: 6471 3266 Email: [OHRPP@nhg.com.sg](mailto:OHRPP@nhg.com.sg)

**11. Statement of Consent**

I have read the above information and had my questions answered. I understand my participation is voluntary and I can withdraw at any time. I agree to participate in this study.

Participant's Name: ____________________________

Participant's Signature: _________________________ Date: ___________

Name of Person Obtaining Consent: _______________________

Signature of Person Obtaining Consent: ___________________ Date: ___________
