# Supplementary material for: Development and feasibility testing of an AI-powered chatbot for early detection of caregiver burden: protocol for a mixed methods feasibility study
Source: Front Psychiatry. 2025 Feb 28;16:1553494. doi: 10.3389/fpsyt.2025.1553494 (PMC11907196; doi:10.3389/fpsyt.2025.1553494)
Supplement: Supplementary file 3 [file DataSheet3.docx]

**Participant Satisfaction Questionnaire for BOTANIC Chatbot**

This questionnaire will be administered at the post-intervention assessment (12 weeks). Please rate your agreement with each statement using the following 5-point scale: 1 = Strongly Disagree 2 = Disagree 3 = Neutral 4 = Agree 5 = Strongly Agree

**1. Perceived Helpfulness**

- BOTANIC helped me become more aware of my stress and burden levels
- The conversations with BOTANIC were helpful in expressing my caregiving experiences
- Using BOTANIC made me feel more supported in my caregiving role
- The chatbot helped me reflect on my caregiving responsibilities

**2. User Experience**

- I felt comfortable sharing my experiences with BOTANIC
- The conversations felt natural and engaging
- I looked forward to interacting with BOTANIC
- The chatbot showed appropriate empathy and understanding

**3. Ease of Use**

- The Telegram interface was convenient to use
- It was easy to start and maintain conversations with BOTANIC
- The response time of the chatbot was satisfactory
- I could easily access previous conversations when needed

**4. Content Quality**

- The questions asked by BOTANIC were relevant to my caregiving experience
- The frequency of interactions was appropriate
- The depth of conversations met my needs
- The chatbot's responses were appropriately tailored to my situation

**5. Technical Aspects**

- The chatbot understood my messages correctly most of the time
- I rarely experienced technical issues while using BOTANIC
- The message notifications were helpful without being intrusive
- The chat history was well-organized and easy to review

**6. Overall Satisfaction**

- Overall, I am satisfied with the BOTANIC chatbot
- I would recommend BOTANIC to other caregivers of ESKD patients
- I would like to continue using BOTANIC after the study ends
- BOTANIC met my expectations as a caregiver support tool

**7. Open-ended Questions**

1. What did you find most valuable about your interactions with BOTANIC?
2. What aspects of BOTANIC could be improved?
3. How did BOTANIC help or hinder your caregiving journey?
4. Were there any topics or concerns you wished BOTANIC could address better?
5. What unexpected benefits or challenges did you experience while using BOTANIC?

**8. Privacy and Trust**

- I felt my personal information was secure when chatting with BOTANIC
- I trusted the chatbot's ability to handle sensitive information
- I was comfortable with how my conversation data was being used
- The privacy policy and data usage were clearly explained

**9. Impact Assessment**

- Using BOTANIC helped me identify signs of caregiver burden early
- The chatbot helped me track changes in my well-being over time
- Interacting with BOTANIC motivated me to seek support when needed
- The regular check-ins helped me maintain awareness of my mental health

**Additional Comments**

Please provide any additional feedback or suggestions about your experience with BOTANIC:

For Research Team Use Only: Participant ID: ____________ Date Completed: ____________ Completion Time: ____________
