# Supplementary material for: Development and feasibility testing of an AI-powered chatbot for early detection of caregiver burden: protocol for a mixed methods feasibility study
Source: Front Psychiatry. 2025 Feb 28;16:1553494. doi: 10.3389/fpsyt.2025.1553494 (PMC11907196; doi:10.3389/fpsyt.2025.1553494)
Supplement: Supplementary file 4 [file DataSheet4.docx]

**Semi-Structured Interview Guide**

**BOTANIC Feasibility Study Post-Intervention Interviews**

**Introduction Script**

"Thank you for participating in the BOTANIC study and agreeing to this interview. We'd like to understand your experience using the chatbot over the past 12 weeks. There are no right or wrong answers - we're interested in your honest feedback. This interview will be audio-recorded and should take about 45-60 minutes. You can skip any questions you don't wish to answer.

**Opening Questions**

1. Could you tell me about your experience as a caregiver?
   - Probe: How long have you been caring for your loved one?
   - Probe: What are your main caregiving responsibilities?

**Experience with BOTANIC**

1. What was it like using the BOTANIC chatbot?
   - Probe: What were your initial expectations?
   - Probe: How did these change over time?
2. Could you walk me through a typical conversation with BOTANIC?
   - Probe: What prompted you to start conversations?
   - Probe: How did the chatbot respond to your concerns?

**Perceived Benefits and Challenges**

1. What aspects of BOTANIC did you find most helpful?
   - Probe: Can you give specific examples?
   - Probe: How did these features help with your caregiving journey?
2. What challenges or difficulties did you encounter?
   - Probe: How did you overcome these challenges?
   - Probe: What support would have been helpful?

**Impact on Caregiver Experience**

1. How did using BOTANIC affect your awareness of caregiver burden?
   - Probe: Did it change how you think about your own well-being?
   - Probe: Did it influence how you seek support?
2. What impact, if any, did BOTANIC have on your caregiving role?
   - Probe: Did it change how you approach caregiving tasks?
   - Probe: Did it affect your relationship with your care recipient?

**Technical Aspects**

1. What was your experience with the technical aspects of BOTANIC?
   - Probe: How was the chatbot's understanding of your messages?
   - Probe: How convenient was the Telegram platform?

**Privacy and Trust**

1. How did you feel about sharing personal information with BOTANIC?
   - Probe: What made you feel comfortable/uncomfortable?
   - Probe: How did you feel about data privacy?

**Suggestions for Improvement**

1. What suggestions do you have for improving BOTANIC?
   - Probe: What features would you add?
   - Probe: What would make it more useful for caregivers?

**Future Implementation**

1. How do you see BOTANIC fitting into caregiver support services?
   - Probe: Would you recommend it to other caregivers?
   - Probe: In what contexts would it be most useful?

**Closing**

1. Is there anything else you'd like to share about your experience with BOTANIC?

**Follow-up Prompts (to be used throughout):**

- Could you tell me more about that?
- Can you give me an example?
- How did that make you feel?
- What do you mean by that?
- Could you explain that further?

**Interview Notes:**

Participant ID: _____________ Date: ___________________ Time Started: _____________ Time Ended: _____________ Interviewer: ______________ Location: ________________

Key Observations:
